# Failure to Replicate a Genetic Association May Provide Important Clues About Genetic Architecture

**DOI:** 10.1371/journal.pone.0005639

**Published:** 2009-06-02

**Authors:** Casey S. Greene, Nadia M. Penrod, Scott M. Williams, Jason H. Moore

**Affiliations:** 1 Department of Genetics, Dartmouth College, Lebanon, New Hampshire, United States of America; 2 Vanderbilt University, Center for Human Genetics, Nashville, Tennessee, United States of America; 3 Department of Community and Family Medicine, Dartmouth Medical School, Lebanon, New Hampshire, United States of America; 4 Department of Computer Science, University of New Hampshire, Lebanon, New Hampshire, United States of America; 5 Department of Computer Science, University of Vermont, Burlington, Vermont, United States of America; 6 Translational Genomics Research Institute, Phoenix, Arizona, United States of America; Institute of Preventive Medicine, Denmark

## Abstract

Replication has become the gold standard for assessing statistical results from genome-wide association studies. Unfortunately this replication requirement may cause real genetic effects to be missed. A real result can fail to replicate for numerous reasons including inadequate sample size or variability in phenotype definitions across independent samples. In genome-wide association studies the allele frequencies of polymorphisms may differ due to sampling error or population differences. We hypothesize that some statistically significant independent genetic effects may fail to replicate in an independent dataset when allele frequencies differ and the functional polymorphism interacts with one or more other functional polymorphisms. To test this hypothesis, we designed a simulation study in which case-control status was determined by two interacting polymorphisms with heritabilities ranging from 0.025 to 0.4 with replication sample sizes ranging from 400 to 1600 individuals. We show that the power to replicate the statistically significant independent main effect of one polymorphism can drop dramatically with a change of allele frequency of less than 0.1 at a second interacting polymorphism. We also show that differences in allele frequency can result in a reversal of allelic effects where a protective allele becomes a risk factor in replication studies. These results suggest that failure to replicate an independent genetic effect may provide important clues about the complexity of the underlying genetic architecture. We recommend that polymorphisms that fail to replicate be checked for interactions with other polymorphisms, particularly when samples are collected from groups with distinct ethnic backgrounds or different geographic regions.

## Introduction

The promise of genome-wide association studies is that they may facilitate discovery of the genetic basis of common human diseases in a hypothesis neutral framework [1], [2]. The technological advances of high-throughput screening combined with database repositories of gene-disease associations provide researchers with an abundance of data for carrying out these extensive studies. The statistical and computational challenges of these studies are considerable. Perhaps the most conspicuous problem lies in multiple testing concerns which arise from the numerous statistical tests performed per dataset leading to a remarkable potential for the discovery of false-positive findings when results are not properly corrected. Pe'er et al. [3] estimate a multiple testing burden of approximately one million tests for genome-wide association analyses in European samples. Even when applied properly, multiple testing corrections do not negate biases inadvertently incorporated into experimental design and data analysis that may also lead to spurious results. In an effort to reduce such spurious associations individual investigators, as well as journal editors, have provided guidelines for conducting genome-wide association studies, advocating replication as a compulsory part of validation [4]. Successful replication provides an important and independent verification of results which helps to prevent the discovery of spurious associations. Unfortunately this replication requirement may filter out real associations when those associations are a part of a larger epistatic interaction or when biology is ignored [5], [6]. Our focus on replication here does not reduce the importance of the discovery phase. A discovery phase with insufficient stringency will likely lower a study's ability to replicate both main effects and interaction effects as the multiple testing penalty at the replication phase is increased.

Intuitively, replication ought to be an effective gold standard for substantiating gene-disease associations because it serves as independent statistical confirmation. Unfortunately reliable replication has not been readily attainable. In a review of genetic association study literature, Hirschhorn et al. [7] reveal that of 166 reported associations, six replicated three or more times. Shriner et al. [5] and Williams et al. [6] also consider the success of association studies in the genome-wide era and discuss the prevalence of findings which, in this genome-wide era, fail to replicate. Many have considered why true associations may not replicate across independent data sets. The predominant explanations account for genetic heterogeneity, environmental interactions, age-dependent effects and inadequate statistical power [8]–[11]. Here we present another likely explanation, gene-gene interactions, as a reason for this non-reproducibility. It is evident that large differences in allele frequency at one interacting locus affect the power to detect the main effect of another locus; however we demonstrate that even small differences in allele frequency at an interacting locus negatively impacts a study's power to replicate a true main effect when the full genetic model is epistatic. Under these circumstances a true association may be lost in an independent sample. The benefit to genome-wide data collection is that the interacting partners may also be measured, providing an opportunity to uncover the full underlying causative models. Conventionally, gene-environment interactions have been recognized as a principal contributing factor to complex disease states. It is also becoming evident that gene-gene interactions are likely to be of critical importance [12]–[14]. Given the likely role of epistasis on human health we must consider what these gene-gene interactions mean for the analysis and interpretation of genome-wide association study results.

Even for genetic models that have the potential to show complete epistasis (i.e. all marginal penetrances are equal), these models only exhibit this characteristic at very specific allele frequencies. In populations with differing allele frequencies, SNPs involved in the interaction may show main effects in addition to epistatic effects. If a small change of allele frequencies at interacting loci can greatly change a study's power to replicate main effects, this may explain why we observe such variability in statistical results from independent samples. For example, with some allele frequencies SNPs in an epistatic model may have equal marginal penetrances (i.e. no main effect). Therefore, methods commonly used in genome-wide studies, such as logistic regression or the chi-square test, will not detect an effect for any of the relevant SNPs. As the allele frequencies deviate from those that are entirely epistatic, one or more SNPs in the model may have main effects detectable by such methods. If these SNPs are found during the discovery phase of a genome-wide association study, replication studies may be undertaken. Depending on the relevant allele frequencies in this new independent sample one of three things may happen: the effect may be replicated, there may be no effect, or the effect may be reversed. Therefore under epistatic models replication of this effect is dependent on the allele frequency at an interacting locus, 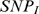, and the epistatic model, neither of which is known in an association study. Here we use simulation to show that a small (<0.1) change in allele frequency at 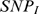 can result in a drastic reduction in the power to replicate the main effect of 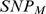 in the context of epistasis.

## Results

**Figure 1:**
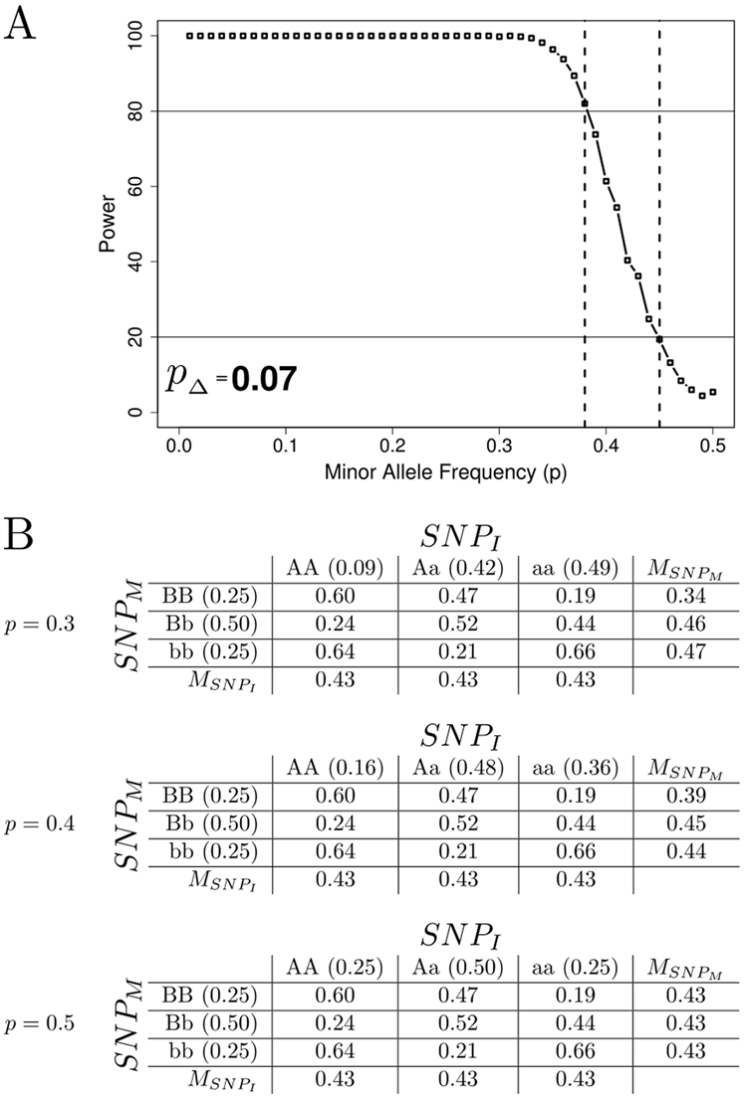
A. This is an example of power results and marginal penetrance tables for an epistatic model with a heritability of 0.1. Part A shows power results. As the minor allele frequency approaches the epistatic minor allele frequency, the power to detect the main effect in a replication sample is reduced. A change of 0.07 in minor allele frequency (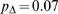) is enough to drop the power to replicate 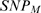 from 80% to 20% for this model. It is apparent in B that as the minor allele frequency, 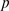, of 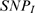 in the sampled population moves from 0.3 to 0.5 the marginal penetrances of the alleles for 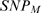 (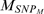) become equal and the main effect is lost. When the replication sample is performed at an allele frequency of 0.3 the power to detect a main effect is near 100%, at an allele frequency of 0.4 the power to detect a main effect is near 60%, and at an allele frequency of 0.5 the marginal penetrances are equivalent and no main effect remains.

We find that a small change in minor allele frequency at 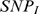 can greatly change a study's power to replicate a main effect at 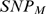. Figure 1A provides an example for a single model at a heritability of 0.1. For this model a change of 0.07 in minor allele frequency at 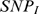 is enough to drop the power to replicate a main effect at 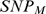 from 80% to 20%. Figure 1B shows how the marginal penetrances for 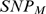 change as the allele frequencies of 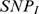 vary. It is this change in allele frequencies at 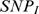 that adjusts the marginal penetrances of 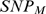 drastically altering the power to replicate a main effect.

**Figure 2:**
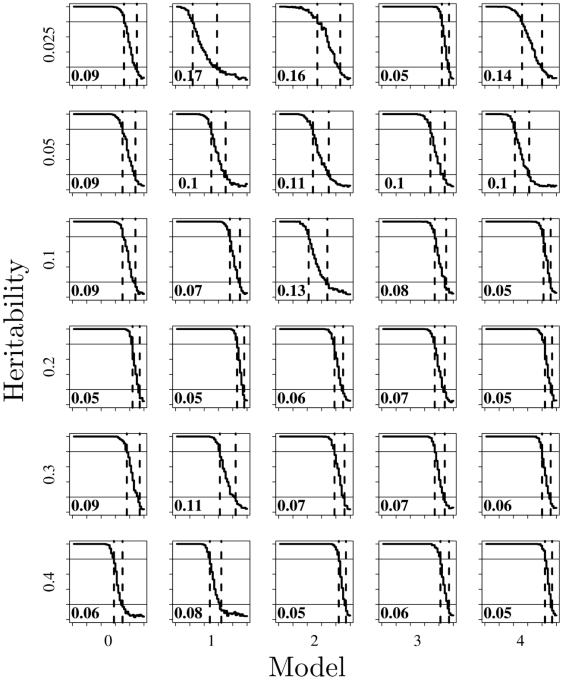
This figure summarizes power for many models and heritabilities. The effect described in Figure 1 is consistent across very large to very small heritability models (0.4 to 0.025). In most cases a change in allele frequency of less than 0.1 is enough to reduce the power to replicate a main effect from 80% to 20%. Results shown are for a sample including 800 cases and 800 controls. Results with datasets containing 400 and 800 individuals are similar and can be found in supplementary figures S1 and S2.

Furthermore we find that across all tested models and heritabilities a similar trend emerges. Figure 2 shows how, for datasets with 1600 individuals and varying heritabilities and models, results remain relatively similar. Each plot in figure 2 is a small version of the plot shown in figure 1A. A relatively small change in allele frequency is all that is required to move from an 80% to a 20% power to replicate an association. This effect is consistent from low (0.025) to high (0.4) broad sense heritabilies due to interaction, although perhaps is more pronounced at higher heritabilities. Still even at the lowest heritability tested for one model a small change in minor allele frequency (0.05) was sufficient to reduce the power from 80% to 20%. This highlights that the change in allele frequency required to prevent replication of a main effect when an underlying epistatic model is responsible is highly dependent on that model. As the true model is unknown in a real world situation, it becomes important to test SNPs which fail to replicate for interactions.

**Figure 3:**
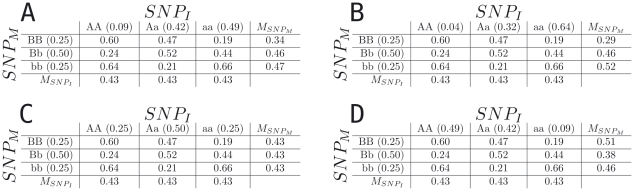
This figure shows marginal penetrances under a number of possible replication scenarios. In part A the marginal penetrances with the allele frequencies found in the discovery phase of a hypothetical genome-wide association study. In part B The marginal penetrances found in the replication phase of a genome-wide association study under a situation where the result would replicate. In part C the marginal penetrances found in the replication phase of a genome-wide association study under a situation where the result would not replicate. In part D the marginal penetrances found in the replication phase of a genome-wide association study under a situation where the allele first discovered as a risk factor would now appear protective.

We also find that, under an epistatic model, an allele that is initially detected as protective can be replicated as a risk factor, even when the model is held constant and only the allele frequencies from which the sample is drawn are varied. Figure 3 shows an example of this effect. The model (i.e. penetrance table) is held constant while the allele frequencies of 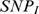 vary. In the discovery phase a main effect is detected (Figure 3A), and an allele, *b*, for 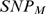 is determined to be a risk allele. In the subsequent replication phase it is possible for that main effect to be confirmed (Figure 3B), no effect to be detected (Figure 3C), or a main effect in the opposite direction may be detected (Figure 3D). In Figure 3D the *b* allele at 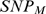 is now protective, reversed from what was found during the discovery phase. This highlights how epistasis can confound replication if only single gene effects are considered.

## Discussion

Epistasis has not yet been widely observed in the æ tiology of complex traits in humans, but interactions are prevalent in model organisms [15], [16]. It seems likely, therefore, that epistasis has not been widely observed in these cases because it is not often investigated [17]. Because results from model organisms suggest that epistasis is likely to be widespread, it would be most prudent to consider the impact of epistasis on experimental design. We have shown that the power to replicate a main effect when the true underlying model is complex depends on the allele frequencies at interacting loci in the replication sample. Furthermore we have shown that even a small change in the allele frequency of 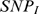 can dramatically reduce a study's power to replicate a main effect for 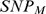. A change in minor allele frequency of less than 0.1 results in a drop in power to replicate from 80% to 20%, even with a heritability as low as 0.025. These dramatic results indicate that a plan for replication must include an analysis of interaction as a contingency when single SNP effects fail to replicate.

Ioannidis et al. [18] examined racial differences in validated markers and discovered that, in 14% of markers studied, there existed large heterogeneity in odds ratios. In a number of cases highlighted by Ioannidis et al. as having heterogeneity in odds ratios in different races, the same allele which is protective in some samples appears as a risk factor in other samples. Here we have shown that this type of replication where effects are significant but in different directions can be explained by allele frequency differences and gene-gene interactions. Because Ioannidis et al. focused only on validated markers this should provide a lower bound on race specific effects, as we have shown that a complex genetic architecture can confound replication and prevent a marker from being considered “validated.” One example of population specific effects is the association of apolipoprotein E (APOE) alleles with dosing of the anticoagulant warfarin. The original study, carried out in Sweden, suggested that individuals homozygous for APOE*E4 required significantly higher doses of warfarin [19]. An independent study in the United Kingdom concluded the APOE*E4 variant played a small but statistically significant role in association with a reduced dosing requirement [20]. When the study was repeated in Italian patients, no correlation was observed and allele frequency differences were cited as a potential explanation for non-replication [21]. More recently, additional attempts were made to relate the APOE*E4 genotype to warfarin dose in a prospective American cohort where approximately 52.2% of patients were Caucasian and showed no association while 47.8% of patients were African American and showed a significantly higher dose requirements in addition to a higher APOE*E4 allele frequency [22].

**Figure 4:**
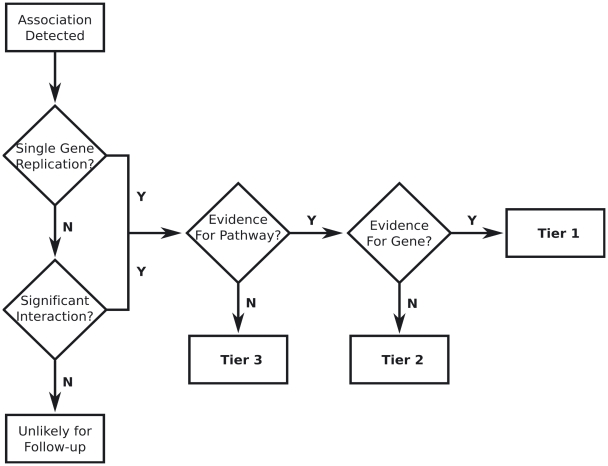
This flowchart represents a method by which candidate SNPs can be divided into tiers for later evaluation based on statistical results and biological information. *Tier 1* markers are likely to provide the easiest gene-function studies but provide the least new information. *Tier 2* markers have the potential to implicate previously unknown genes in known pathways and are also likely to lead to feasible gene-function studies. *Tier 3* markers have the greatest potential to implicate new genes and pathways in the disease process, but gene-function confirmation is likely to be the most difficult for these markers, particularly if clues about role are limited.

Despite the difficulties of replication under a complex model of disease, it is still important to have a strategy for analysis and interpretation of results. Figure 4 presents a flowchart for replication which takes both single gene and interaction results into account and combines these with biological knowledge to divide results into three tiers based on evidence. Under this plan single-SNP associations from an initial sample are examined for similar associations in a replication sample. If these single-SNP effects do not replicate, the SNP in question should be tested in the replication sample for pairwise or higher order interactions with all other SNPs in the dataset (i.e. for analysis of the replication sample, condition on the main effect from the initial sample). This analysis should be performed with care and properly adjusted for multiple comparisons to avoid introducing false positives at this stage. Marchini et al. [23] have shown that, even considering a conservative multiple testing burden, strategies which explicitly examine interactions often have greater power to detect associations when the underlying model includes interactions as this approach can model the true genetic effect and Evans et al. [24] have examined the power of two-stage approaches to detect epistatic interactions. Here, by specifically examining those single-SNPs which had main effects which did not replicate for pairwise interactions the multiple testing burden is further reduced. Markers which do not successfully replicate main effects and which do not have interaction effects in the replication sample are not necessarily without value, but they are less likely candidates for follow-up. It is possible that these markers indicate interaction with a genetic marker unmeasured in the replication sample, a situation particularly likely if few SNPs are genotyped in the replication sample. These markers can also indicate gene-environment interaction, genetic heterogeneity, spurious results, or other complex disease æ tiology. SNPs which possess either single gene or interaction effects in the replication sample are then assessed based on available biological evidence and divided into three tiers. The use of biological knowledge to help guide genome-wide association studies has been advocated in several recent studies [25]–[29]. We suggest biological evidence pertaining to known pathway-disease and gene-disease relationships be considered.

These pathway-disease and gene-disease relationships can be discovered from the literature and prior results via automated tools or manually by reviewing current literature pertaining to the disease, pathways, and genes of interest [30], [31]. The goal here is for the researcher to partition their results into tiers based upon evidence, but to avoid any explicit or implicit valuation of the tiers. The value of markers in each tier is then determined by the objective of the study. Tier one markers are likely to provide the easiest gene-function studies as the polymorphisms are known to exist in a gene related to the disease of interest and are most likely to lead to rapid biomedical applications such as markers for genetic tests, although these results are also the least novel as they implicate both a gene and a pathway already known to be disease associated. Tier two markers are still likely to allow gene-function studies because the gene's mechanism within an involved pathway is known, but they are not as likely to lead to rapid applications. These markers may, however, implicate new genes in disease risk and thus provide new insight as to the disease process. Tier three markers are most likely to be difficult to translate to disease understanding via gene-function studies as they may be in less understood regions of the genome or they may implicate genes in pathways not known to be involved in the disease of interest. These markers allow for the possibility of completely new insight and understanding of the disease of interest but also are the least likely to rapidly translate into biomedical applications. Particularly for these markers, discovery of interactions can provide clues as to their potential mechanisms of action that may facilitate gene-function studies. By dividing association results into tiers, researchers can carefully prioritize SNPs for follow-up based upon the objectives of their research.

Based on these results we suggest that studies of genetic associations should include analysis of interactions, particularly when main effects fail to replicate, and that results, including those from a test of interaction, should be evaluated in a manner that includes pathway-specific and gene-specific information. We present a framework for dividing results into tiers based on statistical and biological information. By dividing results into tiers, researchers can target for follow-up polymorphisms that best meet their predefined objectives. Clearly, association studies which find replicable genetic effects provide valuable insight and biological hypotheses for investigation and in vitro confirmation. It is, however, important to note that studies which detect single SNP associations that then fail to replicate the single-marker effect may still provide important hints as to the the æ tiology of the disease of interest. The ultimate goal is to develop and employ analytical methods that will allow us to assume complexity and scan the entire genome for gene-gene interactions where there might not be detectable main effects in any sample. Only then can we be confident that we are not missing an important component of the genetic architecture of common human diseases.

## Methods

We explored the power to replicate a main effect under an epistatic model. Here we assumed that a main effect had been detected during the discovery phase of a genome-wide association study and simulated replication datasets with various allele frequencies at interacting loci. We examined a study's power to replicate a main effect for a SNP with a true epistatic effect in a highly targeted replication study for a single SNP association. This approach is seen when an investigator attempts to replicate a single SNP from a previous study.

To examine the effects of allele frequency on replication of main effects when there is an underlying complex disease æ tiology we first generated genetic models. These models spanned six broad sense epistatic heritabilities ranging from high to low heritability (0.4, 0.3, 0.2, 0.1, 0.05, and 0.025). For each heritability we generated five models. These models had no main effect when the allele frequencies of the functional SNPs was 0.5. That is, these allele frequencies generated marginal penetrances for each SNP that were equal. Each penetrance function is included in the Supplementary Material S1.

We next generated datasets for each model. To generate datasets with varied allele frequencies that preserve the underlying model, we sampled from populations where the allele frequencies of 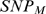 were held constant and the minor allele frequency of 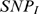 varied from zero to 0.5 in increments of 0.01. For each allele frequency we sampled 500 datasets. Within the populations sampled we insured that all SNP genotypes were in Hardy Weinberg equilibrium at each allele frequency. We used balanced samples with equal numbers of cases and controls consisting of 400, 800, and 1600 individuals. In total we generated 2,295,000 datasets.

We used a chi-square test of independence to assess the significance of associations between SNPs and disease status in these datasets. For purposes of analyzing power we considered 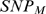 to have been successfully replicated if it had an uncorrected 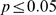. Power, then, is the number of times out of 100 that 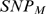 would be found significantly associated in the replication sample (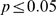) at that allele frequency. This ignored multiple-hypothesis testing issues and represented the situation where there existed a targeted replication hypothesis. This approach specifically considered the power to replicate an association discovered in a detection sample in an independent replication sample. Application of a Bonferroni correction to these results yielded, as expected, an overall decrease in the power to replicate a main effect. The change in allele frequency at 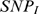 required to reduce power to replicate the main effect at 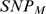 did not significantly change and thus these results are not presented. These results are available from the authors upon request.

## Supporting Information

Figure S1 The effect described in Supplementary Figure S1 is consistent across very large to very small heritability models (0.4 to 0.025). In most cases a change in allele frequency of less than 0.1 is enough to reduce the power to replicate a main effect from 80% to 20%. Results shown are for a sample including 200 cases and 200 controls.
(0.86 MB EPS)

Figure S2 The effect described in Supplementary Figure S2 is consistent across very large to very small heritability models (0.4 to 0.025). In most cases a change in allele frequency of less than 0.1 is enough to reduce the power to replicate a main effect from 80% to 20%. Results shown are for a sample including 400 cases and 400 controls.
(0.89 MB EPS)

Supplementary Material S1 Supplementary Tables
(0.02 MB PDF)
