# Supplementary material for: Failure to Replicate a Genetic Association May Provide Important Clues About Genetic Architecture
Source: PLoS One. 2009 Jun 2;4(6):e5639. doi: 10.1371/journal.pone.0005639 (PMC2685469; doi:10.1371/journal.pone.0005639)
Supplement: Supplementary Material S1 — Supplementary Tables (0.02 MB PDF) [file pone.0005639.s003.pdf]

Table 1: Penetrance values for epistasis model 1 at a heritability of 0.025

	AA	Aa	aa
BB	0.26	0.15	0.06
Bb	0.18	0.13	0.18
bb	0.01	0.21	0.20

Table 2: Penetrance values for epistasis model 2 at a heritability of 0.025

	AA	Aa	aa
BB	0.05	0.21	0.07
Bb	0.17	0.09	0.20
bb	0.14	0.16	0.08

Table 3: Penetrance values for epistasis model 3 at a heritability of 0.025

	AA	Aa	aa
BB	0.05	0.22	0.05
Bb	0.15	0.10	0.19
bb	0.20	0.12	0.11

Table 4: Penetrance values for epistasis model 4 at a heritability of 0.025

	AA	Aa	aa
BB	0.06	0.00	0.01
Bb	0.00	0.04	0.00
bb	0.01	0.00	0.06

Table 5: Penetrance values for epistasis model 5 at a heritability of 0.025

	AA	Aa	aa
BB	0.21	0.26	0.05
Bb	0.24	0.14	0.25
bb	0.09	0.23	0.23

Table 6: Penetrance values for epistasis model 1 at a heritability of 0.05

	AA	Aa	aa
BB	0.47	0.23	0.27
Bb	0.24	0.27	0.42
bb	0.24	0.44	0.09

Table 7: Penetrance values for epistasis model 2 at a heritability of 0.05

	AA	Aa	aa
BB	0.18	0.03	0.20
Bb	0.08	0.18	0.02
bb	0.11	0.06	0.22

Table 8: Penetrance values for epistasis model 3 at a heritability of 0.05

	AA	Aa	aa
BB	0.40	0.18	0.37
Bb	0.13	0.37	0.25
bb	0.46	0.21	0.25

Table 9: Penetrance values for epistasis model 4 at a heritability of 0.05

	AA	Aa	aa
BB	0.12	0.23	0.35
Bb	0.22	0.30	0.11
bb	0.36	0.10	0.36

Table 10: Penetrance values for epistasis model 5 at a heritability of 0.05

	AA	Aa	aa
BB	0.06	0.24	0.32
Bb	0.36	0.19	0.11
bb	0.08	0.24	0.31

Table 11: Penetrance values for epistasis model 1 at a heritability of 0.1

	AA	Aa	aa
BB	0.40	0.30	0.38
Bb	0.22	0.48	0.20
bb	0.54	0.12	0.60

Table 12: Penetrance values for epistasis model 2 at a heritability of 0.1

	AA	Aa	aa
BB	0.60	0.47	0.19
Bb	0.24	0.52	0.44
bb	0.64	0.21	0.66

Table 13: Penetrance values for epistasis model 3 at a heritability of 0.1

	AA	Aa	aa
BB	0.01	0.46	0.33
Bb	0.56	0.22	0.25
bb	0.13	0.35	0.43

Table 14: Penetrance values for epistasis model 4 at a heritability of 0.1

	AA	Aa	aa
BB	0.40	0.15	0.10
Bb	0.05	0.31	0.15
bb	0.31	0.04	0.41

Table 15: Penetrance values for epistasis model 5 at a heritability of 0.1

	AA	Aa	aa
BB	0.41	0.67	0.27
Bb	0.53	0.53	0.42
bb	0.54	0.29	0.91

Table 16: Penetrance values for epistasis model 1 at a heritability of 0.2

	AA	Aa	aa
BB	0.76	0.40	0.25
Bb	0.51	0.31	0.68
bb	0.03	0.79	0.20

Table 17: Penetrance values for epistasis model 2 at a heritability of 0.2

	AA	Aa	aa
BB	0.29	0.09	0.85
Bb	0.45	0.32	0.21
bb	0.12	0.58	0.04

Table 18: Penetrance values for epistasis model 3 at a heritability of 0.2

	AA	Aa	aa
BB	0.25	0.53	0.65
Bb	0.79	0.31	0.54
bb	0.13	0.80	0.22

Table 19: Penetrance values for epistasis model 4 at a heritability of 0.2

	AA	Aa	aa
BB	0.40	0.70	0.52
Bb	0.78	0.37	0.79
bb	0.36	0.88	0.20

Table 20: Penetrance values for epistasis model 5 at a heritability of 0.2

	AA	Aa	aa
BB	0.67	0.96	0.07
Bb	0.70	0.54	0.87
bb	0.59	0.61	0.85

Table 21: Penetrance values for epistasis model 1 at a heritability of 0.3

	AA	Aa	aa
BB	0.06	0.80	0.02
Bb	0.48	0.21	0.78
bb	0.66	0.46	0.10

Table 22: Penetrance values for epistasis model 2 at a heritability of 0.3

	AA	Aa	aa
BB	0.60	0.06	0.74
Bb	0.07	0.62	0.15
bb	0.72	0.16	0.42

Table 23: Penetrance values for epistasis model 3 at a heritability of 0.3

	AA	Aa	aa
BB	0.25	0.94	0.65
Bb	0.90	0.47	0.94
bb	0.72	0.91	0.25

Table 24: Penetrance values for epistasis model 4 at a heritability of 0.3

	AA	Aa	aa
BB	0.38	0.56	0.06
Bb	0.57	0.14	0.72
bb	0.05	0.72	0.07

Table 25: Penetrance values for epistasis model 5 at a heritability of 0.3

	AA	Aa	aa
BB	0.43	0.23	0.58
Bb	0.05	0.60	0.21
bb	0.94	0.03	0.47

Table 26: Penetrance values for epistasis model 1 at a heritability of 0.4

	AA	Aa	aa
BB	0.04	0.83	0.23
Bb	0.85	0.17	0.73
bb	0.18	0.75	0.23

Table 27: Penetrance values for epistasis model 2 at a heritability of 0.4

	AA	Aa	aa
BB	0.06	0.95	0.28
Bb	0.92	0.26	0.81
bb	0.34	0.78	0.34

Table 28: Penetrance values for epistasis model 3 at a heritability of 0.4

	AA	Aa	aa
BB	0.88	0.03	0.27
Bb	0.05	0.55	0.06
bb	0.21	0.08	0.82

Table 29: Penetrance values for epistasis model 4 at a heritability of 0.4

	AA	Aa	aa
BB	0.94	0.04	0.88
Bb	0.05	0.71	0.44
bb	0.87	0.45	0.14

Table 30: Penetrance values for epistasis model 5 at a heritability of 0.4

	AA	Aa	aa
BB	0.29	0.09	0.90
Bb	0.06	0.59	0.13
bb	0.95	0.10	0.22
